# Asymptomatic loss of intraepidermal nerve fibers with preserved thermal detection thresholds after repeated exposure to severe cold

**DOI:** 10.1002/brb3.917

**Published:** 2018-02-04

**Authors:** Thomas Krøigård, Martin Wirenfeldt, Toke K. Svendsen, Søren H. Sindrup

**Affiliations:** ^1^ Department of Neurology Odense University Hospital Odense C Denmark; ^2^ Department of Clinical Research Faculty of Health Science University of Southern Denmark Odense C Denmark; ^3^ Department of Pathology Odense University Hospital Odense C Denmark

**Keywords:** cold‐induced peripheral neuropathy, healthy subject, intraepidermal nerve fiber density, nerve conduction studies, quantitative sensory testing

## Abstract

**Background:**

Cold‐induced peripheral neuropathy has been described in individuals exposed to severe cold resulting in pain, hypersensitivity to cold, hyperhidrosis, numbness, and skin changes. Nerve conduction studies and thermal detection thresholds are abnormal in symptomatic patients, and intraepidermal nerve fiber density (IENFD) in skin biopsies is reduced.

**Case presentation:**

A 41‐year‐old male was included as a healthy subject in a study of the spontaneous variability of quantitative sensory testing (QST), nerve conduction studies (NCS), and IENFD. Unexpectedly, IENFD was significantly reduced, whereas the rest of the examination was normal except for reduced vibration detection threshold. The results were confirmed at follow‐up examination. The subject had been repeatedly exposed to severe cold resulting in short lasting numbness and paresthesia while living in the eastern part of Greenland and the northern part of Norway.

**Conclusion:**

Loss of intraepidermal nerve fibers caused by exposure to severe cold may be asymptomatic, and their function assessed by thermal detection thresholds may be preserved. This case illustrates that QST and IENFD are complementary tests and that subclinical cold‐induced peripheral neuropathy may be prevalent in subjects living in or near polar regions which could have implications for the recruitment of healthy subjects.

## 1 INTRODUCTION

Cold‐induced peripheral neuropathy is a well‐known complication of nonfreezing or freezing cold injury resulting in pain, hypersensitivity to cold, hyperhidrosis, numbness, and skin changes in affected areas (Schafer & Thompson, 1995). It has been characterized in patients living in the northern parts of Scandinavia demonstrating neurophysiological changes resulting in reduced motor and sensory conduction velocities in the lower extremities, increased distal motor latency in the lower extremities, and reduced sensory conduction velocity in the upper extremities in patients with persistent cold intolerance (Arvesen, Wilson, & Rosen, 1996). Furthermore, it has been shown that thermal detection thresholds are impaired in the fingers of patients with previous frostbites (Burström et al., 2017). Very recently, it was shown that the density of intraepidermal nerve fibers in skin biopsies was reduced in 90% of 42 patients with symptoms of chronic cold‐induced peripheral neuropathy (Vale et al., 2017). In this cohort, nerve conduction studies were normal in all patients.

## 2 CLINICAL PRESENTATION

**Figure 1:**
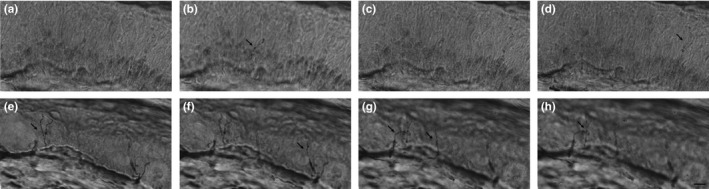
Photomicrographs at successive focal planes of 50‐μm sections stained with anti‐PGP9.5 antibodies to visualize the intraepidermal nerve fibers (arrows) in skin biopsies. The subject exposed to severe cold (A–D) had a reduced number of intraepidermal nerves, which appeared fragmented. A skin biopsy from a healthy subject (45‐year‐old male, E–H) analyzed at the same time in our laboratory had several intraepidermal nerves at different focal planes. Scale bar: 10 μm

A 41‐year‐old male was examined as a healthy subject in a prospective study on the spontaneous variation in epidermal nerve fiber density, quantitative sensory testing, and nerve conduction studies. He fulfilled the inclusion criteria which comprised no symptoms of peripheral neuropathy and no risk factors for developing peripheral neuropathy such as excessive alcohol consumption, diabetes, hypothyroidism, connective tissue disease, with no use of medications known to affect the peripheral nervous system and no previous lesions to the nervous system. The subject had no comorbidity and did not use any medications on a regular basis. Neurological examination was normal except for decreased sensibility for vibration at the first toe bilaterally. Study procedures were performed at baseline examination and repeated after 7 weeks in June and August 2016 after written informed consent was obtained. They consisted of quantitative sensory testing (QST), standard nerve conduction studies (NCS), and skin biopsies for quantification of the intraepidermal nerve fiber density (IENFD). QST included evaluation of cold detection threshold, warmth detection threshold, heat pain threshold, mechanical detection threshold, mechanical pain threshold, and vibration detection threshold performed according to DFNS standards (Rolke et al., 2005). For NCS, peroneal nerve distal motor latency, motor conduction velocity, compound motor action potential amplitude, and F‐wave latency, sural nerve sensory conduction velocity and sensory nerve action potential amplitude, and tibial nerve distal motor latency, compound motor action potential amplitude, and F‐wave latency were measured bilaterally. The reference values obtained at Glostrup University Hospital, Denmark (personal communication), were used for classification. Skin biopsies were obtained from the distal leg and processed according to EFNS and PNS guidelines (Joint Task Force of the EFNS and the PNS, 2010). The linear density, IENFD, was determined using bright field immunohistochemistry after staining with anti‐PGP 9.5 antibodies. IENFD was classified as reduced when it was below the 0.05 quantile of the comprehensive normative data (Lauria et al., 2010). At baseline examination, we found reduced IENFD and vibration detection threshold, whereas the rest of the examination was normal, including thermal detection thresholds (Table 1). A representative section of the skin biopsy is presented in Figure 1. The results were confirmed at the seven‐week follow‐up examination.

**Table 1:** Quantitative sensory testing (QST), nerve conduction studies (NCS), and intraepidermal nerve fiber density (IENFD)

	Baseline	7 weeks	Reference values^a^
QST			
Cold detection threshold (°C from baseline)	−2, 2	−5, 0	−0, 7—−12, 6
Warmth detection threshold (°C from baseline)	4, 8	5, 0	2, 3—15, 8
Heat pain threshold (°C)	44, 4	45, 8	41, 8—50
Mechanical detection threshold (mN)	4, 3	6, 5	0, 4—41, 1
Mechanical pain threshold (mN)	21, 1	48, 5	15, 3—695, 4
Vibration detection threshold	3, 8	3, 2	5, 2—8
NCS			
Peroneal nerve			
Distal motor latency (ms)	4, 8	4, 7	<5, 5
Motor conduction velocity (m/s)	43, 7	41, 0	>40, 4
Amplitude of compound motor action potential (mV)	3, 7	3, 6	>3, 5
F‐wave latency (ms)	56, 4	56, 1	<58, 4
Sural nerve			
Sensory conduction velocity (m/s)	49, 1	49, 1	>45, 1
Amplitude of sensory nerve action potential (mV)	6	7, 2	>5, 4
Tibial nerve			
Distal motor latency (ms)	3, 3	3, 2	<5, 2
Amplitude of compound motor action potential (mV)	13, 9	20, 2	>7, 4
F‐wave latency (ms)	58, 4	57, 9	<59, 8
IENFD (fibers/mm)	2, 0	2, 7	>4, 4

a Age‐group specific.

On repeated evaluation of the subjects medical history, he disclosed that he had been exposed to extreme cold while living in the eastern part of Greenland between 2000 and 2002, the northern part of Norway between 2005 and 2006, and during annual recreational trips lasting one or 2 weeks to the northern parts of Scandinavia since 2003. In Greenland, he had spent the night in tents at −40°C approximately 10 times. On approximately five occasions, he had noted numbness and paresthesia in the distal parts of the toes lasting less than 2 weeks. On one occasion, the subject had suffered a severe frostbite to the distal part of the fingers on one hand. He confirmed that he had no sensory symptoms at the time of examination.

## 3 DISCUSSION

Unexpectedly, we found that intraepidermal nerve fiber density was significantly reduced in a healthy subject who had experienced repeated exposures to severe cold. We cannot exclude that the subject had a reduced intraepidermal nerve fiber density prior to the exposure to cold and that it is a nonspecific finding. Intraepidermal nerve fiber density can be reduced for several reasons including metabolic and genetic conditions. In this study, we excluded that the patient had diabetes or hypothyroidism. The reduced vibration detection threshold supports the conclusion that a subclinical lesion to the peripheral nerves was in fact present. On this basis, we found that the subject has a subclinical cold‐induced peripheral neuropathy. This case illustrates that examination of the intraepidermal nerve fiber density and quantitative sensory testing are complementary tests evaluating the structure and function of the peripheral nervous system, respectively. Examination of warmth detection thresholds is a surrogate for the function of C‐fibers, which were the fibers quantified in skin biopsies. Loss of intraepidermal nerve fibers may be asymptomatic and sensory function unaffected if the remaining nerve fibers are intact. In small fiber neuropathy, reduced IENFD was found to be more sensitive than abnormal QST (Devigili et al., 2008), but in a significant proportion of patients with normal IENFD QST was abnormal suggesting that a multimodal approach is needed for the diagnosis of small fiber neuropathy. Addition of the quantitative sudomotor axon reflex test further increases the diagnostic yield in small fiber neuropathy (Thaisetthawatkul, Fernandes Filho, & Herrmann, 2013). Abnormal sense of vibration at the toe suggests lesion to the very distal parts of the myelinated nerve fibers or the sensory end‐organs (Pacinian corpuscles) in the skin. The normal nerve conduction studies and thermal detection thresholds in our subject, which is in contrast to the previous studies (Arvesen et al., 1996; Burström et al., 2017), may reflect reduced sensitivity of these tests and does not exclude the lesion of large or small nerve fibers. Loss of intraepidermal nerve fibers may be a more sensitive test of peripheral nerve damage caused by exposure to cold than quantitative sensory testing, but in the study of 42 patients with chronic cold‐induced peripheral neuropathy, loss of function was demonstrated for both small and large fiber functions in approximately 60%–80% of patients (Vale et al., 2017). Quantitative sensory testing, however, was not used as a diagnostic tool, but rather to assess the somatosensory phenotype.

Furthermore, this case exemplifies the difficulties regarding the case definition of a “healthy subject” in clinical studies, that is, the extent of history, signs, and objective measures necessary to make sure that the subject has no relevant neurological disease (Gierthmühlen et al., 2015).
